# A multicenter randomized controlled trial of esophagectomy with or without prophylactic supraclavicular node dissection: a phase 3 trial (JCOG2013, MODERN3)

**DOI:** 10.1093/jjco/hyad071

**Published:** 2023-06-28

**Authors:** Shigeru Tsunoda, Yasuhiro Tsubosa, Keita Sasaki, Ryunosuke Machida, Ryosuke Kita, Haruhiko Fukuda, Kazuo Koyanagi, Hiroya Takeuchi, Takashi Kamei, Shinji Mine, Kazuhiro Noma, Ken Kato, Yuko Kitagawa, the Japan Esophageal Oncology Group of Japan Clinical Oncology Group

**Affiliations:** Department of Surgery, Graduate School of Medicine, Kyoto University, Kyoto; Division of Esophageal Surgery, Shizuoka Cancer Center, Shizuoka; JCOG Data Center/Operations Office, National Cancer Center Hospital, Tokyo; JCOG Data Center/Operations Office, National Cancer Center Hospital, Tokyo; JCOG Data Center/Operations Office, National Cancer Center Hospital, Tokyo; JCOG Data Center/Operations Office, National Cancer Center Hospital, Tokyo; Department of Gastroenterological Surgery, Tokai University School of Medicine, Isehara; Department of Surgery, Hamamatsu University School of Medicine, Hamamatsu; Department of Surgery, Tohoku University Graduate School of Medicine, Sendai; Department of Esophageal and Gastroenterological Surgery, Juntendo University Graduate School of Medicine, Tokyo; Department of Gastroenterological Surgery, Okayama University Graduate School of Medicine, Okayama; Gastrointestinal Medical Oncology Division, National Cancer Center Hospital, Tokyo; Department of Surgery, Keio University School of Medicine, Tokyo, Japan

**Keywords:** esophageal neoplasms, supraclavicular node, phase III, esophagectomy

## Abstract

The need for prophylactic supraclavicular lymph node dissection during esophagectomy with radical lymphadenectomy remains controversial. A randomized phase III trial was launched in August 2022 to confirm the non-inferiority of esophagectomy with D2 lymphadenectomy except for supraclavicular lymph node dissection to standard D2 lymphadenectomy in terms of overall survival for patients with resectable upper or middle thoracic esophageal cancer. This study will enroll 480 patients from 54 Japanese institutions over 5 years. The primary endpoint includes overall survival, and the secondary endpoints include relapse-free survival, perioperative and late complication incidences, supraclavicular lymph node recurrence, salvage cervical treatment incidence, synchronous cervical and abdominal procedure proportion, operation time and the number of operating surgeons. This trial has been registered at the Japan Registry of Clinical Trials under study number jRCT1030220248.

## Teaser

A randomized phase III trial aimed to confirm the non-inferiority of esophagectomy with D2 lymphadenectomy except for supraclavicular lymph node dissection to standard D2 lymphadenectomy for patients with resectable thoracic esophageal cancer.

## Introduction

Esophageal cancer is the sixth leading cause of cancer-related death worldwide (1). The mainstay of treatment for resectable esophageal cancer is esophagectomy with regional lymphadenectomy, unless endoscopic resection is indicated (2–5). However, the extent of the resection differs between the East, including Japan and the West. The supraclavicular lymph nodes (SCLNs) were regarded by the Japanese classification as regional nodes (2,3), while outside of the regional nodes by the Union for International Cancer Control staging system (6), and their involvement as distant metastasis in Western countries.

Esophagectomy with radical lymphadenectomy, including upper mediastinal and cervical nodal dissection, was established in Japan in the 1980s (7,8). The literature compared ‘Three-field dissection’ with ‘two-field dissection’ in the 1990s. However, their definition and range were inconsistent among the studies. Two randomized studies compared ‘three-field’ and ‘two-field’ dissection, but both failed to reveal the survival benefit of ‘three-field’ lymph node dissection (9,10). One study was conducted from 1987 to 1993 in Japan and the other from 2013 to 2016 in China, and both studies were upfront open esophagectomy without neoadjuvant therapy, which is completely different from the current practice of neoadjuvant treatment followed by minimally invasive esophagectomy.

Among the cervical node stations, the cervical paraesophageal node (No. 101) and supraclavicular node (No. 104) are defined as the regional nodes for thoracic esophageal cancer in Japan (11). The lymphatic chain involvement along the recurrent laryngeal nerve (RLN), consisting of the cervical paraesophageal node (No. 101) and RLN node (No. 106rec) with no anatomical clear border (12), is reported at 40% (7). Therefore, the cervical paraesophageal node (No. 101) dissection is important. Conversely, the incidence of pathological metastasis of supraclavicular node (No. 104) of upper and middle esophageal cancer patients who had been diagnosed with no supraclavicular node involvement was only 4% from a recent three-arm phase III trial, JCOG1109 (13,14), comparing cisplatin plus 5-FU (CF) versus docetaxel, cisplatin plus 5-FU (DCF) versus radiotherapy with CF as preoperative therapy for locally advanced esophageal cancer (unpublished data). The recent meta-analysis that compared esophagectomy with SCLN dissection versus esophagectomy without SCLN dissection, which both completed cervical paraesophageal node dissection, did not reveal the survival merit of prophylactic SCLN dissection (15).

A broader cervical incision is required for bilateral SCLN dissection and may cause laryngeal penetration or aspiration (16). The surgery arm of JCOG0502 (17) revealed a trend of higher incidence of overall postoperative complications in patients who underwent prophylactic supraclavicular dissection compared with patients who did not (48.1 vs. 38.3%) (18). Routine prophylactic SCLN dissection may not be essential because postoperative SCLN metastasis can often be managed by either surgery or chemoradiotherapy (19,20) at the time of relapse.

Therefore, this clinical trial aimed to confirm the non-inferiority of esophagectomy with D2 lymphadenectomy, except for SCLN, to standard D2 lymphadenectomy in terms of overall survival (OS) for patients with resectable upper or middle thoracic esophageal cancer.

**Figure 1:**
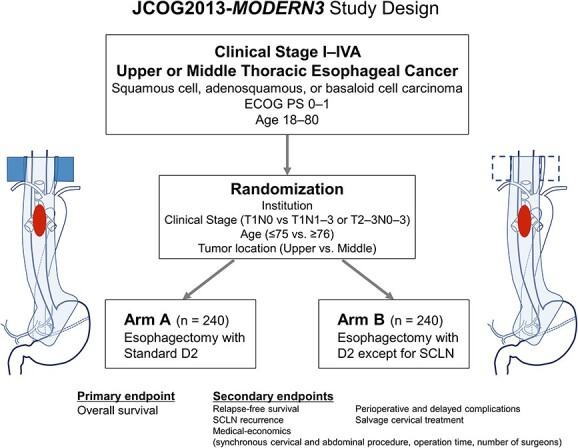
The study schema of JCOG2013-*MODERN3.*

This study was approved by the Protocol Review Committee of the Japan Clinical Oncology Group (JCOG) in March 2022, and centralized approval was obtained from the institutional review board of the National Cancer Center Hospital in May 2022. Patient enrollment began in August 2022. The schematics of JCOG2013 are shown in Fig. 1.

## Protocol digest of the JCOG2013

### Objectives

This trial aims to confirm the non-inferiority of esophagectomy with D2 lymphadenectomy, except for SCLN, to standard D2 lymphadenectomy in terms of OS for patients with resectable upper or middle thoracic esophageal cancer.

### Study setting

A multi-institutional, two-arm, open-label, randomized phase III trial.

### Endpoints

The primary endpoint of this study includes OS in all randomized patients. OS is defined as the number of days between randomization and death from any cause, and it is censored on the last day the patient is alive. The secondary endpoints include relapse-free survival, perioperative and late complication incidences, SCLN recurrence incidences, salvage cervical treatment incidence, synchronous cervical and abdominal procedure proportion, operation time and the number of operating surgeons.

### Eligibility criteria

#### Inclusion criteria

Patients fulfilling all the following criteria are eligible for this study:

1. Histologically proven squamous cell carcinoma, adenosquamous carcinoma or basaloid carcinoma.

2. Primary tumor located in the upper or middle thoracic esophagus, and all lesions including intramural metastasis, intraepithelial extension and secondary lesion located in the thoracic esophagus; concomitant secondary lesion in the cervical esophagus indicative of endoscopic treatment is allowed.

3. Esophageal cancer is clinically staged as I, II, III or IVA (except for T4) following the Union for International Cancer Control, eighth edition, of the TNM Classification of malignant tumors (6). One or two CF courses or three or fewer DCF courses are allowed, in cases of neoadjuvant chemotherapy, but preoperative chemoradiotherapy is not allowed, and the clinical stage before neoadjuvant chemotherapy must be I, II, III or IVA (except for T4).

4. Aged 18–80 years.

5. Eastern Cooperative Oncology Group performance status of 0 or 1.

6. One-stage trans-right-thoracic esophagectomy, either open or minimally invasive, including a robot-assisted approach, is indicated for R0 resection.

7. No previous history of chemotherapy, radiotherapy and immunotherapy for other cancer. Previous history of endocrine therapy for breast cancer or prostate cancer is allowed.

8. No previous anterior neck surgery or surgery for right lung cancer.

9. No previous treatment of esophageal cancer except for (i)–(iii).

(i) Non-curative primary tumor resection by endoscopic mucosal resection (EMR)/endoscopic submucosal dissection (ESD).

(ii) Neoadjuvant chemotherapy of one or two courses of CF or three or fewer courses of DCF for clinical stages I (T1N1), II, III or IVA (except for T4) esophageal cancer patients.

(iii) Curative resection achieved by EMR/ESD against metachronous multiple cancer.

10. Sufficient organ functions.

(i) White blood cell of ≥3000/mm^3^.

(ii) Platelet of ≥100 000/mm^3^.

(iii) Total bilirubin of <1.5 mg/dl.

(iv) Aspartate transaminase of ≤100 IU/l.

(v) Alanine aminotransferase of ≤100 IU/l.

(vi) Creatinine of ≤1.5 mg/dl.

(vii) SpO_2_ of ≥95% (room air)

11. Written informed consent.

#### Exclusion criteria

Patients with any of the following criteria are ineligible for this study:

1. Synchronous or metachronous (within 5 years) malignancies except for carcinoma *in situ* or mucosal tumors curatively treated with local therapy.

2. Active infection requiring systemic therapy.

3. Body temperature of ≥38°C.

4. Female during pregnancy, within 28 days of post parturition or during lactation; males who want their partners to be pregnant.

5. Psychological disorders difficult to participate in this clinical study.

6. Receiving continuous systemic corticosteroid or immunosuppressant treatment.

7. Poorly controlled diabetes mellitus with continuous use of insulin or hypoglycemic agents.

8. Uncontrolled arterial hypertension.

9. History of unstable angina pectoris within 3 weeks or myocardial infarction within 6 months before registration.

10. Uncontrolled valvular disease, dilated cardiomyopathy and hypertrophic cardiomyopathy.

11. Severe emphysema, interstitial pneumonia or pulmonary fibrosis based on chest computed tomography (CT).

### Randomization

Registration with the planned thoracic (thoracotomy/thoracoscopy/robot-assisted surgery) and abdominal approach (laparotomy/laparoscopy/robot-assisted surgery) declaration is made using a web-based system to the JCOG Data Center after confirming the fulfillment of the eligibility criteria. Patients are randomized to either arm A (standard D2) or arm B (D2 except for SCLN) by the minimization method with a random component balancing the arms with the institution, clinical stage (I [T1N0M0] vs. I [T1N1M0], II, III, IVA), age (≤75 vs. ≥76) and tumor location (upper thoracic vs. middle thoracic).

### Treatment methods

Surgical resection of the esophagus and reconstruction should be performed at one time. Planned two-stage esophagectomy is not allowed; however, intraoperative conversion is accepted to ensure patient safety. Details of the operative method are as follows:

1. Operative approach.

(i) Thoracic approach (both arms)

Esophagectomy is performed via a right thoracotomy (with a skin incision of >5 cm) in the lateral decubitus position or a right thoracoscopy, including robot assistance (with a skin incision of ≤5 cm) in the lateral decubitus or prone positions. Operation only via a mediastinoscopy is not allowed. Intraoperative biopsy or cytology, as well as bronchial artery and thoracic duct preservation are not specified.

(ii) Abdominal approach (both arms)

Open or laparoscopic, including hand- or robot-assistance approach is performed. Hand-assisted laparoscopy is classified as a laparoscopic approach. Splenectomy is only allowed for assurance of hemostasis in case of bleeding.

2. Lymph node dissection.

D2 lymphadenectomy according to the 11th edition of the Japanese classification of esophageal cancer (11) is performed in arm A. No.1, 2, 3a, 7, and 110 lymph node dissection is mandated in upper thoracic disease. The aforementioned D2 lymphadenectomy except for SCLN is performed in arm B. A right cervical incision can be omitted in arm B if the right cervical paraesophageal node (No. 101R) dissection is completed in the thoracic procedure. Sentinel lymph node dissection is not allowed.

3. Reconstruction method (both arms).

Based on the standard of each participating institution, the reconstruction route and organs used for reconstruction are selected. Anastomosis is performed via neck incision. The method of anastomosis is not specified.

### Quality control of surgery

1. Certified surgeon for esophagectomy via right thoracotomy.

Surgeons who have experienced ≥10 open esophagectomies (OEs) and who are credentialed by the Study Chair to perform OE as an operator or leading assistant.

2. Certified surgeon for esophagectomy via right thoracoscopy.

The study-specific quality assurance (QA) committee of surgery, which is credentialed by the Study Chair, certifies the surgeon with sufficient thoracoscopic surgical skills using a video of thoracoscopic esophagectomy (TE). Certified surgeons should perform TE as an operator or leading assistant. Both of the following criteria of a certified surgeon must be fulfilled.

(i) Experienced ≥20 TEs.

(ii) Certification by either the QA committee of surgery in the Japan Esophageal Oncology Group (JEOG) or the Japan Society for Endoscopic Surgery (21) in esophageal cancer.

3. Certified surgeon for esophagectomy via right robot-assisted thoracoscopy.

Certified surgeons should perform robotic esophagectomy (RE) as an operator. All of the following criteria of a certified surgeon must be fulfilled.

(i) Experienced ≥20 TEs.

(ii) Certification for TE by either the study-specific QA committee of surgery in JEOG or the Japan Society for Endoscopic Surgery in the area of esophageal cancer.

(iii) Experienced ≥10 REs.

(iv) Proctors qualified by the Japan Society for Endoscopic Surgery in the area of esophageal cancer or certification by the QA committee of surgery in JEOG.

4. Intraoperative photographs.

The QA committee of surgery performs a central peer review of the surgical procedure for all cases using intraoperative photos of upper mediastinal and cervical lymphadenectomy.

### Follow-up

All randomized patients are followed up for at least 5 years after completing patient enrollment. Tumor markers (carcinoembryonic antigen and squamous cell carcinoma) are evaluated at least every 3 months for the first year, and every 6 months from the second to fifth years. Contrast-enhanced CT for the cervix, chest, abdomen and pelvis is evaluated at least every 6 months for the first 5 years. Endoscopy is evaluated annually.

### Study design and statistical analysis

This randomized trial has been designed to confirm the non-inferiority of esophagectomy with D2 lymphadenectomy, except for SCLN (arm B), to standard D2 lymphadenectomy (arm A) in terms of OS for patients with resectable upper or middle thoracic esophageal cancer. The primary analysis is to be conducted 3 years after enrollment completion. The hazard ratio between treatment arms and its confidence interval, estimated by the Cox proportional hazard model stratified by clinical stage (I [T1N0M0] vs. I [T1N1M0], II, III and IVA), age (≤75 vs. ≥76 years) and tumor location (upper thoracic vs. middle thoracic) is used to test the non-inferiority of the arm B in terms of OS. A total of 147 events would be required with a one-sided α of 0.05, power of 70%, an expected accrual period of 5 years, a follow-up period of 3 years and a non-inferiority margin of 6% at 3-year OS, assuming the expected 3-year OS rates for arm A and arm B are 80 and 81%, respectively. Non-inferiority will be concluded if the upper limit of the confidence interval of the hazard ratio does not exceed the limit of 1.35, which is the non-inferiority margin. The required sample size of 456 patients is necessary to observe 147 events according to the Schoenfeld and Richter’s method (22). The total sample size was set at 480 patients to account for patients lost to follow-up. All statistical analyses will be conducted at the JCOG Data Center.

### Interim analysis and monitoring

Two interim analyses are planned taking multiplicity into account using the Lan–DeMets method with the O’Brien and Fleming type alpha spending function (22). The first interim analysis will be conducted after enrolling half of the planned number of patients. The second interim analysis will be conducted after completing the planned patient enrollment and protocol treatment. The Data and Safety Monitoring Committee of the JCOG will independently review the interim analysis reports from the group investigators and group statistician. The trial will be terminated at the first interim analysis if arm B is superior to arm A in terms of OS with an adjusted significance level, and at the second interim analysis if arm B is non-inferior to arm A in terms of OS with an adjusted significance level. Regarding futility termination, the trial will be stopped if arm B is inferior to arm A in terms of OS with a point estimate of the hazard ratio of >1.35. In-house monitoring will be performed every 6 months by the JCOG Data Center to evaluate and improve study progress, data integrity and patient safety.

## jRCT registration number

This trial has been registered at the Japan Registry of Clinical Trials as jRCT1030220248 [https://jrct.niph.go.jp/latest-detail/jRCT1030220248].

### Participating institutions (from north to south)

Hokkaido University, Iwate Medical University, Tohoku University, Fukushima Medical University, Tochigi Cancer Center, Saitama Cancer Center, Saitama Medical University International Medical Center, National Cancer Center Hospital East, Chiba University, National Canter Center Hospital, Tokyo Medical University, Tokyo Women’s Medical University, Keio University, Showa University, Tokyo Medical and Dental University, Cancer Institute Hospital, Toranomon Hospital, Juntendo University, Tokai University, Kanagawa Cancer Center, Kitasato University, Niigata Cancer Center, Niigata University, Gifu University, Shizuoka Prefectural General Hospital, Hamamatsu University School of Medicine, Shizuoka Cancer Center, Aichi Cancer Center, Nagoya University, Kyoto University, Osaka University, Osaka International Cancer Institute, National Hospital Organization Osaka Medical Center, Osaka General Medical Center, Osaka City General Hospital, Osaka Medical and Pharmaceutical University, Kansai Medical University, Kobe University, Kansai Rosai Hospital, Hyogo Cancer Center, Himeji Japanese Red Cross Society Himeji Hospital, Okayama University, Kawasaki Medical School, Hiroshima University, Hiroshima City Asa Hospital, Yamaguchi University, National Hospital Organization Shikoku Cancer Center, Kochi Medical Center, National Hospital Organization Kyushu Cancer Center, Kyushu University, Saga University, Kumamoto University, Oita University, Kagoshima University.
